# L1 Makes Immunological Progress by Expanding Its Relations

**DOI:** 10.1155/1998/23451

**Published:** 1998

**Authors:** Guni Kadmon, Anthony M. P. Montgomery, Peter Altevogt

**Affiliations:** 1 Department of Cellular Immunology 0710 German Cancer Research Centre Im Neuenheimer Feld 280 Heidelberg D-69120 Germany; 2 Department of Immunology The Scripps Research Institute 10666 North Torrey Pines Rd. La Jolla California 92037 USA

**Keywords:** Adhesion, integrins, immune system, L1, lymphocyte, nervous system

## Abstract

The cell-adhesion molecule L1 was originally described in the nervous system. It has recently been detected in CD4^+^ T lymphocytes, peripheral B lymphocytes, and granulocytes in the human immune system and in similar leucocyte types in the murine immune system. L mediates neural recognition by Ca^+2^, Mg^+2^-independent homophilic binding. In the human and murine immune systems, L1 binds to the “classical” vitronectin receptor, *α*V*β*3, and fibronectin receptor, *α*5*β*1, respectively, and abstains from homophilic binding. Homophilic L1 binding probably involves antiparallel alignment of several interactive domains. Integrin binding is mediated by a short segment of immunoglobulinlike domain 6, which includes two RGD repeats in rodent L1 and one RGD motif in human L1. L1 is modulated in activated leucocytes *in vitro* in parallel to L-selectin, and diverse cell types release intact L *in vivo* and *in vitro*. Released L1 can bind to laminin and adheres to the extracellular matrix of sciatic nerve, M21 melanoma, and possibly spleen and other tissues. It can support integrin-dependent cell migration and preliminary data implicate it in tumor development and transnodal lymphocyte migration.


*Developmental Immunology*, 1998, Vol. 6, pp. 205–213
Reprints available directly from the publisher
Photocopying permitted by license only

© 1998 OPA (Overseas Publishers Association)
N.V. Published by license under the
Harwood Academic Publishers imprint,
part of the Gordon and Breach Publishing Group
Printed in Malaysia

### *Minireview*

### L1 Makes Immunological Progress by Expanding Its Relations

GUNI KADMON[a], ANTHONY M.P. MONTGOMERY[b] and PETER ALTEVOGT[a*]

[a]*Department of Cellular Immunology 0710, German Cancer Research Centre, Im Neuenheimer Feld 280, D-69120 Heidelberg, Germany*; [b]*Department of Immunology, The Scripps Research Institute, 10666 North Torrey Pines Rd., La Jolla, California 92037*

(*Received 15 August 1996; In final form 12 April 1997*)

The cell-adhesion molecule L1 was originally described in the nervous system. It has recently been detected in $CD4^+$ T lymphocytes, peripheral B lymphocytes, and granulocytes in the human immune system and in similar leucocyte types in the murine immune system. L1 mediates neural recognition by $Ca^{+2}$, $Mg^{+2}$-independent homophilic binding. In the human and murine immune systems, L1 binds to the "classical" vitronectin receptor, $\alpha V\beta 3$, and fibronectin receptor, $\alpha 5\beta 1$, respectively, and abstains from homophilic binding. Homophilic L1 binding probably involves antiparallel alignment of several interactive domains. Integrin binding is mediated by a short segment of immunoglobulinlike domain 6, which includes two RGD repeats in rodent L1 and one RGD motif in human L1. L1 is modulated in activated leucocytes *in vitro* in parallel to L-selectin, and diverse cell types release intact L1 *in vivo* and *in vitro*. Released L1 can bind to laminin and adheres to the extracellular matrix of sciatic nerve, M21 melanoma, and possibly spleen and other tissues. It can support integrin-dependent cell migration and preliminary data implicate it in tumor development and transnodal lymphocyte migration.

*Keywords:* Adhesion, integrins, immune system, L1, lymphocyte, nervous system

### INTRODUCTION

The development of a functional cellular immune system and effective immune response depends on selective leucocyte trafficking to, from, and within organs of the immune system as well as to variable but specific addresses such as inflammatory sites. Correct leucocyte trafficking is mediated by contact-dependent interactions between leucocytes and post-capillary endothelial cells, glandular stroma cells, and extracellular matrices. These interactions require multiple-adhesion mechanisms that can recognize appropriate targets and be modulated by the cell during recognition events. Intensive investigations of

*Corresponding author.

TABLE I L1-Expressing Cell Types of Haematopoietic Lineages in Man and Mouse

| Human | Murine |
| --- | --- |
| $CD4^+$ peripheral blood *T lymphocytes* | Mature *thymocytes* |
| ≤20% $CD31^+$ peripheral blood *T lymphocytes* | Approx. 20% of $CD3^+$ splenic *T lymphocytes* |
| $CD19^+$ peripheral blood *B lymphocytes* | $B220^+$ splenic *B lymphocytes* |
| $CD14^+$ peripheral blood *monocytes* | $GR1^+$ bone marrow *granulocytes* |
| *Tumors*: haematopoietic tumor lines including *monocytic*—U937, HL60; *T lymphoma*—MOLT-3, Jurkat; *B lymphoma*—Raji. *Neuroblastomas*; *Melanoma*—M21; *small cell lung tuimors.* | *Tumors*: several haematopoietic tumor lines, neural tumors. |

leucocyte extravasation have produced ample information on the mechanisms mediating leucocyte-endothelial recognition (for extensive reviews, see Butcher, 1991; Imhof and Dunon, 1995). There is, however, little information on the adhesion mechanisms that mediate leucocyte migration through and beyond the endothelial wall, through lymph glands and inflamed tissues.

During the last year, the cell-adhesion molecule L1 has emerged as an important candidate for mediating such processes. L1 is primarily expressed by neural and blood cells involved in targeted migration and is at present the only known cell-adhesion molecule that is abundant in both nervous and immune systems. Its importance in human ontogenesis is underlined by the severity of neurological disorders associated with mutations in the L1 gene. These include hydrocephalus and mental retardation and have recently been united under the acronym CRASH syndromes (Fransen et al., 1995). Associated manifestations in the immune system remain to be defined.

In the nervous system, L1 is predominantly expressed by neuronal subsets and Schwann cells. It mediates neuronal adhesion, axonal navigation and fasciculation, axon-Schwann cell recognition, and possibly granule-cell migration. In the immune system, L1 is expressed by neutrophils and lymphocyte subsets (Table I). Its function(s) in the immune system is not certain, but preliminary studies using tumor lines, T and B lymphoblasts suggest that L1 may be involved in both leucocyte-leucocyte and leucocyte-endothelial cell interactions.

L1 is a transmembranal glycoprotein with apparent molecular weight of 190-230 kD. Its protein backbone contains six immunoglobulin domains (domains Ig 1-6), five fibronectin type III domains (domains $FN_{III}1$-5), and two known alternatively spliced mini-exons, exons 2 and 27 (Figure 1A). Murine and rat L1 share 97% identical amino acids (Miura et al., 1991), human and murine L1 approximately 87% (Kobayashi et al., 1991), and the related molecules, chicken NgCAM and *Drosophila* neuroglian have 43% (Burgoon et al., 1991) and 27% (Bieber et al., 1989) amino acid identity to rodent L1, respectively. The glycosylation pattern of L1 is variable and accounts for 20-35% of its apparent molecular weight. L1 variably expresses the carbohydrate epitopes HNK-1 and L3 (Fig. 1B). The HNK-1 epitope is involved in L1 binding to laminin (Hall et al., 1993) and ligand binding to P- and L-selectin (Needham and Schnaar, 1993; Sammar et al., 1994), whereas the L3 glycan has been implicated in L1-NCAM cohesion in neurones (Horstkorte et al., 1993). The expression of the HNK-1 and L3 epitopes by L1 in blood cells has not been studied.

### L1 LIGANDS

The functional vicissitude of L1 has been attributed to its recently discovered diversity of binding mechanisms (Table II). In rodents and man, L1 can bind to at least three counterreceptors with distinct cation requirements. The major L1 ligand for L1-expressing murine leucocytes and lymphoma ESb-MP cells appears to be the "classical" fibronectin receptor, $\alpha5\beta1$ integrin (Ruppert et al., 1995). L1-$\alpha5\beta1$ binding requires $Ca^{+2}$, $Mg^{+2}$ and preactivation of

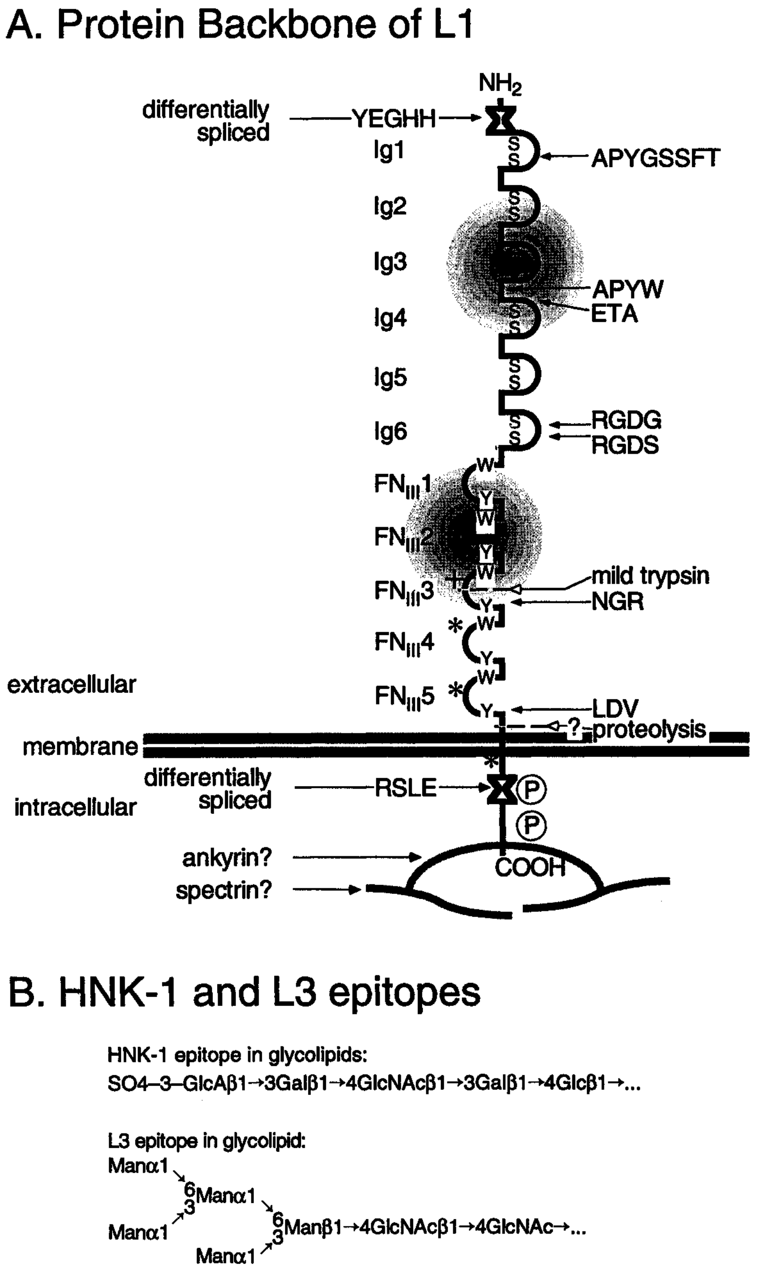

FIGURE 1 Schematic representation of L1. (A) L1 is a transmembranal glycoprotein with six immunoglobulinlike (Ig1-6) and five fibronectin type III-like ($FN_{III}$1-5) domains in its extracellular segment. Two proteolytic sites (broken lines) generate characteristic fragments (vertical double arrows) *in vivo* and *in vitro*. Alternative splicing of two microexons generates several isoforms. In neurones, the intracellular tail can possibly be anchored to spectrin via a neural isoform of ankyrin. The positions of potential $\alpha5\beta1$ (ETA, RGDG, RGDS, NGR), $\alpha4\beta1$ (LDV), and FGF receptor-homology sequences (APY, APYW), basic box (+), cystein residues outside (*) and inside (S) Ig domains, and conserved tyrosine (Y) and tryptophan (W) residues in $FN_{III}$ domains are indicated. Clouded areas denote putative interactive segments mediating homophilic binding. (B). The glycan structure of HNK-1- and L3-immunoreactive glycolipids. L1 variably expresses both epitopes. HNK-1- and L3-immunoreactive glycolipids inhibit L1 binding to laminin and NCAM, respectively. HNK-1-immunoreactive glycolipids also bind to P-and L-selectins.

TABLE II Homophilic Binding and Integrin Binding by L1 in Different Cell Types Analyzed by Direct Binding and Antibody Perturbation Studies

| Species | Probe cells or beads | L1expression | Examined cell interaction:aggregation or adhesion | Bindingmechanism |
|---|---|---|---|---|
| Mouse | Neurones | Yes | Adhesion to immobilized L1 | L1-L1+L1-other? |
| | Neuroblastoma N2A cells | Yes | Short-term homotypic aggregation | L1-L1 |
| | Neuroblastoma N2A cells | Yes | Long-term homotypic aggregation | L1-$\alpha5\beta1$? |
| | Microbeads coated with N2A L1 | Yes | Homotypic aggregation | L1-L1 |
| | Microbeads coated with N2A L1 | Yes | Adhesion to thrombocytes ($L1^-$) | L1-$\alpha5\beta1$ |
| | Microbeads coated with N2A L1 | Yes | Adhesion to bEnd3 endothelioma ($L1^-$) | L1-unknownL1-$\alpha$V? |
| | Activated thymocytes | Yes | Adhesion to immobilized L1 | $\alpha5\beta1$-L1 |
| | ESb-MP cells (nonactivated) | Yes | Short-term homotypic aggregation | None |
| | ESb-MP cells (activated) | Yes | Short-term homotypic aggregation | L1-$\alpha5\beta1$ |
| | Bone marrow cells | Yes | Adhesion to thrombocytes ($L1^-$) | L1-$\alpha5\beta1$+L1-other? |
| Man | Melanoma M21 cells | Yes | Adhesion to immobilized human & rat L1 | $\alpha$V$\beta3$-L1 |
| | Melanoma M21 cells | Yes | Adhesion to heat-denatured L1 | $\alpha$V$\beta3$-L1 |
| | J558L cells transfected with L1 gene from M21 cells | Yes | Adhesion to immobilized L1 | L1-L1 |
| | J558L cells transfected with L1 gene from M21 cells | Yes | Adhesion to immobilized heat-denatured L1 | None |
| | Lymphoblastic leukemia MED-B1 | No | Adhesion to immobilized human & murine L1 | $\alpha$V$\beta3$-L1 |

$\alpha5\beta1$ via CD24 antibodies, by $Mn^{+2}$ cations, or by phorbolesters. In human cells of haematopoietic lineages (Ebeling et al., 1996) and melanoma M21 cells (Montgomery et al., 1996), L1 strongly binds to $\alpha V\beta3$ and weakly to $\alpha5\beta1$. The binding between L1 and $\alpha V\beta3$ requires $Ca^{+2}$ and $Mg^{+2}$ but no prior activation of the cells, although $Mn^{+2}$ may enhance the binding.

In the nervous system, L1 mediates neuronal recognition by temperature- and cation-independent homophilic binding (Lemmon et al., 1989). It is at present not certain that neuronal L1 recognizes additional ligands. Binding between neuronal L1 and integrins has to our knowledge not been examined under conditions that support integrin-L1 interactions. However, purified L1 from murine neuroblastoma N2A cells binds murine $\alpha5\beta1$ and serves as a good substrate for $\alpha5\beta1$-mediated leucocyte adhesion in the presence of $Mn^{+2}$ *in vitro* (Ruppert et al., 1995). Purified L1 from rat pheochromocytoma PC12 cells (NILE) interacts with human $\alpha V\beta3$ and possibly with $\alpha5\beta1$ (Montgomery et al., 1996). There are at present no indications that L1 binds to mammalian homologues of the chicken neuronal adhesion molecules axonin-1 or F11, the chondroitin sulphate proteoglycan neurocan, or the presumptive astrocytic ligand(s) that is recognized by the chicken homologue of L1, NgCAM (for a review, see Brümmendorf and Rathjen, 1993).

### BINDING MECHANISMS OF L1

### Homophilic Binding

Holm et al. (1995) have reported that microbeads coated with intact L1 or with fusion proteins derived from L1 domains Ig3-4 or $FN_{III}3$-5 strongly adhere to substrate-immobilized L1. Microbeads coated with fusion proteins derived from other short segments of L1 or from longer segments that include these domains underwent homotypic aggregation but weaker or no adhesion to substrate-immobilized L1. These observations appear to indicate that mutual attraction among parallel domains of L1 could facilitate L1 clustering on the cell surface, but *trans* binding between L1 molecules may primarily involve domains Ig3-4 and $FN_{III}2$-4 (Figure 2A). Systematic analysis of the homophilic binding mechanism of L1 is still wanting. However, the data are consistent with the proposed model for the homophilic binding mechanisms of carcinoembryonic antigen (CEA; Zhou et al., 1993) and platelet-endothelial celladhesion molecule (PECAM-1, CD31; Fawcett et al., 1995). Like L1, CEA and CD31 belong to the immunoglobulin supergene family and function as $Ca^{+2}$-independent cell-adhesion molecules. Homophilic CEA binding involves double reciprocal interactions between the amino terminal and an internal domain. Homophilic CD31 binding is primarily mediated by domains 2, 3, 5, 6, but requires the presence of all six domains. It has therefore been suggested that stable homophilic binding is mediated by antiparallel alignment of apposing molecules and correct interdigitation of corresponding "high-affinity" domains (Fig. 2A).

### Heterophilic Binding to $\alpha V\beta 3$ and $\alpha 5\beta 1$ Integrins

The major integrin-binding site of L1 lies in a segment of domain Ig6 that includes the consensus integrin-binding motif arginine-glycine-aspartate (RGD; Fig. 2B and Fig. 2C). The adhesion of human melanoma M21 cells to substrate-immobilized L1 depends on $\alpha$V expression and can be inhibited by antibodies to $\alpha V\beta 3$. It can also be prevented by **RGDG → DVDG** mutation in domain Ig6 of substrate-adsorbed human L1 (Montgomery et al., 1996). Moreover, the synthetic peptide CW**RGD**GRDLQELGDSDKYFIE**DGR**LV derived

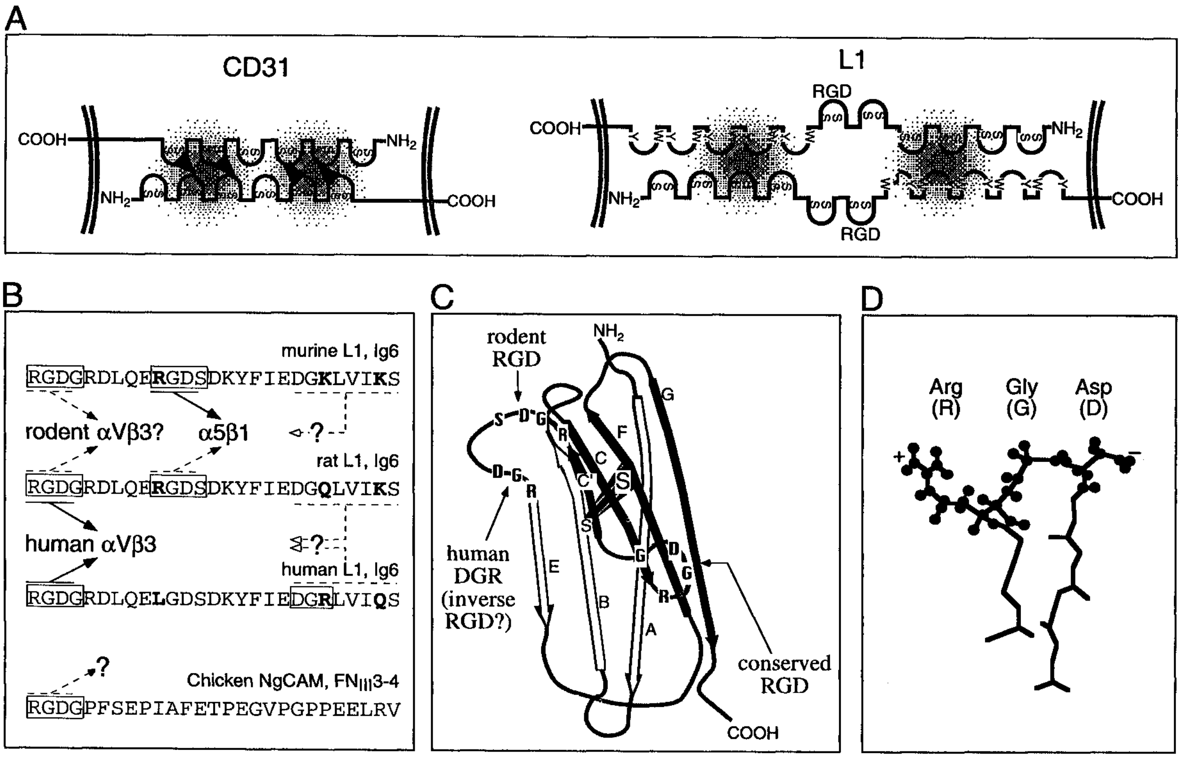

FIGURE 2 Presumptive binding mechanisms of L1. (A) Model for homophilic binding by CD31 and L1. Binding occurs between interdigitated high-affinity domains (clouded regions) and requires correct antiparallel alignment of the intact molecules. (B) Presumptive differential $\alpha V\beta 3$- and $\alpha 5\beta 1$-binding RGD motifs (boxed) in domain Ig6 of rodent and human L1. The homologous integrin-binding peptides (single-letter notation) of rodent and human L1 and the nonhomologous RGD-containing segment of chicken NgCAM are shown. (Boldface type) nonconserved residues. (Solid closed arrows) probable binding based on experimental evidence. (Broken closed arrows) possible, but unverified binding. (Broken open arrows) nonconserved segments that possibly influence selective binding. (C) Schematic model of domain Ig6 of L1. B-strands, disulphide bridge (S—S), and putative positions of RGD sequences are marked. Modified from Williams (1987) by alignment with C2-set domains with known crystal structure. (D) Wire-frame model of the RGD loop required for $\alpha 1\beta 1$, $\alpha 2\beta 1$, and possibly $\alpha 5\beta 1$ binding. Atom positions (heavy dots) in the RGD residues are shown. Acidic (−) and basic (+) side chains are denoted by clouded shading. Adopted from Haas and Plow, 1994.

from domain Ig6 of human L1 predominantly binds to $\alpha V\beta 3$ and supports the adhesion of $\alpha V\beta 3$-expressing MED-B1 cells but not $\alpha 5\beta 1$-expressing Nalm-6 cells (Ebeling et al., 1996). On the other hand, the synthetic peptide CW**RGD**GRDLQE**RGDS**DK derived from domain Ig6 of murine L1 binds to $\alpha 5\beta 1$, inhibits the homotypic aggregation of murine lymphoma ESb-MP cells, and supports $\alpha 5\beta 1$-dependent adhesion of murine peripheral thymocytes (Ruppert et al., 1995).

By amino acid sequence comparisons between domain Ig6 of L1 and immunoglobulinlike domains with known crystal structure, domain Ig6 of L1 most closely resembles domain Ig2 of VCAM-1. It potentially has a C2-set immunoglobulin fold (Fig. 2C) with a short C′ strand and particularly elongated C′-E loop. Rodent and human L1 contain a conserved RGD motif (RGDS; Fig. 2B) in the putative C-C′ loop. A second RGD motif (RGDS; Fig. 2B) is expressed by rodent L1 in the putative C′-E loop. However, crystallographic analysis has not been performed, and it cannot be excluded that both RGD motifs are expressed on the same $\beta$ loop (C-C′ or C-D). In human L1, arginine to leucine change disrupts the RGDS motif in the C′-E loop, whereas a lysine to arginine change generates an "inverse" RGD (DGR) motif at a different position, presumably on the same $\beta$ loop (Fig. 2C).

### Mechanisms of Selective Integrin Binding

Since L1 is expressed by similar neuronal and leucocyte subsets in mouse and man, the existence of variable RGD sites in the otherwise highly conserved molecule may reflect evolutionary adaptation of L1 to binding functionally analogous integrins in different species. The tripeptidyl RGD is expressed by most integrin-binding matrix proteins and serves as the main binding site for most integrin-substrate adhesion molecules. However, different integrin dimers bind to different RGD-expressing proteins, indicating that the molecular context is a determinant factor in selective RGD recognition (for reviews, see Ruoslahti, 1988; Haas and Plow, 1994). RGD recognition by $\alpha 1\beta 1$ and $\alpha 2\beta 1$ depends on RGD presentation in a flexible conformation at the apex of a type II $\beta$ loop with the basic and acidic side chains of arginine and aspartate pointing away from one another (Fig. 2D). A$5\beta 1$ recognizes cyclic RGD-containing peptides, suggesting similar conformational constraints, whereas $\alpha V\beta 3$ binds both to cyclic and linear RGD-containing peptides (for a review, see Haas and Plow, 1994). A$5\beta 1$ strongly binds to murine (Ruppert et al., 1995) but not human (Ebeling et al., 1996) L1. Therefore, $\alpha 5\beta 1$ possibly recognizes the fibronectinlike motif RGDS of rodent L1 (Fig. 2B) that is missing in human L1. AV$\beta 3$ appears to bind to the conserved RGDG site of L1 (Fig. 2B), since M21 cell adhesion to human L1 is abolished by mutation of this motif (Montgomery et al., 1996), and human $\alpha V\beta 3$ mediates cell binding to human, rat (Montgomery et al., 1996), and murine L1. However, binding of $\alpha V\beta 3$ to L1 has so far not been detected in murine cells. It is therefore likely that species-specific differences in the binding properties of $\alpha V\beta 3$ and possibly $\alpha 5\beta 1$ codetermine the choice of L1 ligand. It is at present an open issue whether amino acid changes to the carboxyl terminal to the RGD motifs of L1 (Fig. 2B) are also important to selective integrin-L1 binding.

In addition to conformational constraints on RGD presentation, conjoint non-RGD binding sites in the ligand may also contribute to selective ligand recognition by RGD-binding integrins. Binding of $\alpha V$ integrin(s) to collagen is enhanced in the presence of short noncollagen RGD peptides, indicating that RGD recognition activates cryptic collagen-binding sites in $\alpha V$ integrins (Agrez et al., 1991). Binding of $\alpha 5\beta 1$ to the RGD motif in domain $FN_{III}10$ ("cell-binding domain") of fibronectin is imperative to stable contact between $\alpha 5\beta 1$ and matrix-embedded fibronectin. Yet, the affinity of $\alpha 5\beta 1$ to fibronectin is strongly reduced by mutations in domains $FN_{III}8$ and $FN_{III}9$ of fibronectin (Obara et al., 1988; Aota et al., 1991), suggesting that additional non-RGD integrin-recognition sites are also important. Interestingly, L1 shares with domains $FN_{III}8$ and $FN_{III}9$ of fibronectin several non-RGD peptide sequences that have been implicated in $\alpha 5\beta 1$ binding (Fig. 1). Preliminary studies from our laboratory indicate that the synthetic cycloheptapeptide ***RRETAWA***, resembling one of those sites, interferes with L1-$\alpha 5\beta 1$ binding through action on a non-RGD site of L1. However, additional studies will be required to determine the significance of this observation and whether L1 domains other than domain Ig6 indeed participate in regulating the integrin-binding potential of L1.

### Possible Roles of Homophilic and Integrin Binding in L1-dependent Adhesion, Migration, and Signal Transduction

L1 joins a growing number of cellular receptors for "classical" matrix-binding integrins (for a review, see Imhof and Dunon, 1995). Among these, CD31 is of particular interest since L1 and CD31 appear to have similar homophilic binding mechanisms (Fig. 2A) and high affinity for $\alpha V\beta 3$ (Piali et al., 1995; Montgomery et al., 1996) but are expressed by mutually exclusive lymphocyte subsets (Ebeling et al., 1996). The functional significance of these dual binding mechanisms is not clear. However, it seems possible that homophilic L1 binding primarily mediates cell-cell recognition and stimulation of FGF receptor-mediated signal transduction, whereas L1-integrin binding may support cellular locomotion. L1-transfected J558L cells that adhere to immobilized L1 by homophilic binding undergo very limited movement on substrate L1. Conversely, L1-expressing M21 cells interact with L1 via $\alpha V\beta 3$ and exhibit pronounced $\alpha V\beta 3$-dependent haptotactic movement on substrate L1 (Montgomery et al., 1996). In addition, monoclonal L1 antibody 324 that specifically inhibits L1-integrin interactions does not perturb the short-term aggregation of murine cerebellar neurones and neuroblastoma N2A cells (Rathjen and Schachner, 1984). But it inhibits the migration of granule-cell neurones (Lindner et al., 1983) and neurite elongation (Fischer et al., 1986) in cerebellar explants from postnatal mice. Moreover, homophilic L1 binding activates FGF receptor-dependent signal transduction in rat neurones, but probably not nonreceptor-type tyrosine kinases (Doherty et al., 1995). However, neurite extension by murine cerebellar neurones on immobilized L1 is specifically abolished by homozygous *src* deletion (Ignelzi et al., 1994), and L1 cross-linking by antibody 324 modulates $pp60^{c\text{-}src}$-dependent phosphorylation in neurones (Atashi et al., 1992).

### L1 RELEASE AND INTERACTION WITH THE EXTRACELLULAR MATRIX

Several types of neurones release increased amounts of L1 in response to nerve growth factor *in vitro* (Richter-Landsberg et al., 1984). Melanoma M21 cells spontaneously release L1 at an average rate of approximately $10^4$ molecules/cell/hour (Montgomery et al., 1996), and lymphocytes downregulate L1 expression upon activation *in vitro* and *in vivo* (Hubbe et al., 1993). Stimulation of neutrophils with the phorbolester PMA leads to rapid loss of L1 from the cell surface with remarkably similar kinetics to the shedding of L-selectin. L-selectin shedding has recently been shown to depend on an MMP1-like metalloendoprotease (Preece et al., 1996). Whether a similar mechanism also applies to L1 remains to be examined. Released L1 can be detected in association with the extracellular matrix of murine sciatic nerve (Martini and Schachner, 1986), and within intratumor laminin strands of human melanoma M21 (Montgomery et al., 1986). Selective binding between purified L1 and laminin has been demonstrated (Hall et al., 1993), but the physiological function of matrix-embedded L1 is still uncertain. Conceivably, matrix-embedded L1 gradients may locally promote targeted cell migration as a substrate for integrin adhesion. Recent experiments using recombinant L1 have shown that L1 indeed can support integrin-mediated cell migration (Duzcmal et al., 1997). The possibility that released L1 may promote intratumor angiogenesis by interaction with $\alpha V$ integrins of sprouting vessels can also be considered.

### CONCLUSIONS

The cell-adhesion molecule L1 serves both as a cell-adhesion molecule and as a matrix-embedded substrate for cell migration. In both functions, L1 may undergo homophilic interaction or bind to "classical"

integrin-matrix receptors. Dual homophilic and integrin binding has also been reported for the nonneural recognition molecules CD31 and E-cadherin. Homophilic binding has primarily been associated with early recognition events. Binding between homophilic cell-adhesion molecules and integrins appears to stabilize cell connections and promote cell motility. This information implies that the major difference between cell-cell and cell-matrix interactions pertain to early recognition and choice of contact. But the mechanisms mediating long-term adhesion and migration on cellular and matrix substrates may be related.

### *References*

Agrez M.V., Bates R.C., Boyd A.W., and Burns G.F. (1991). Arg-Gly-Asp-containing peptides expose novel collagen receptors on fibroblasts: Implications for wound healing. Cell Regul. **2**: 1035-1044.

Aota S.-i., Nagai T., and Yamada K.M. (1991). Characterization of regions of fibronectin besides the arginine-glycine-aspartic acid sequence required for adhesive function of the cell-binding domain using site-directed mutagenesis. J. Biol. Chem. **266**: 15938-15943.

Atashi J.R., Klinz S.G., Ingraham C.A., Matten W.T., Schachner M., and Maness P.F. (1992). Neural cell adhesion molecules modulate tyrosine phosphorylation of tubulin in nerve growth cone membranes. Neuron **8**: 831-842.

Bieber A.J., Snow P.M., Hortsch M., Patel N.H., Jacobs J.R., Traquina Z.R., Schilling J., and Goodman C.S. (1989). Drosophila neuroglian: A member of the immunoglobulin superfamily with extensive homology to the vertebrate neural cell adhesion molecule L1. Cell **59**: 447-460.

Brümmendorf T., and Rathjen F.G. (1993). Axonal glycoproteins with immunoglobulin- and fibronectin-type III-related domains in vertebrates: Structural features, binding activities, and signal transduction. J. Neurochem. **61**: 1207-1219.

Burgoon M.P., Grumet M., Mauro V., Edelman G.M., and Cunningham B.A. (1991). Structure of the chicken neuron-glia cell adhesion molecule, Ng-CAM: Origin of polypeptides and relation to Ig superfamily. J. Cell Biol. **112**: 1017-1029.

Butcher E.C. (1991). Leukocyte-emdothelial cell recognition: Three (or more) steps to specificity and diversity. Cell **67**: 1033-1036.

Doherty P., Williams E., and Walsh F.S. (1995). A soluble chimeric form of the L1 glycoprotein stimulates neurite outgrowth. Neuron **14**: 57-66.

Duczmal A., Schöllhammer S., Katich S., Ebeling O., Schwartz-Albiez R., and Altevogt P. (1997). The L1 adhesion molecule supports $\alpha V\beta 3$-mediated migration of human tumor cells and activated T lymphocytes. Biochem. Biophys. Res. Commun. **232**: 236-239.

Ebeling O., Duczmal A., Aigner S., Geiger C., Schöllhammer S., Kemshead J.T., Möller P., Schwartz-Albiez R., and Altevogt P. (1996). L1 adhesion molecule on human lymphocytes and monocytes: Expression and involvement in binding to $\alpha V\beta 3$ integrin. Eur. J. Immunol. **26**: 2508-2516.

Fawcett J., Buckley C., Holness C.L., Bird I.N., Spragg J.H., Saunders J., Harris A., and Simmons D.L. (1995). Mapping the homotypic binding sites in CD31 and the role of CD31 adhesion in the formation of interendothelial cell contacts. J. Cell Biol. **128**: 1229-1241.

Fischer G., Künemund V., and Schachner M. (1986). Neurite outgrowth patterns in cerebellar microexplant cultures are affected by antibodies to the cell surface glycoprotein L1. J. Neurosci. **6**: 605-612.

Fransen E., Lemmon V., Van-Camp G., Vits L., Coucke P., and Willems P.J. (1995). CRASH syndrome: Clinical spectrum of corpus callosum hypoplasia, retardation, adducted thumbs, spastic paraparesis and hydrocephalus due to mutations in one single gene, L1. Eur. J. Hum. Genet. **3**: 273-284.

Haas T.A., and Plow E.F. (1994). Integrin-ligand interactions: A year in review. Curr. Opin. Cell Biol. **6**: 656-662.

Hall H., Liu L., Schachner M., and Schmitz B. (1993). The L2/HNK-1 carbohydrate mediates adhesion of neural cells to laminin. Eur. J. Neurosci. **5**: 34-43.

Holm J., Appel F., and Schachner M. (1995). Several extracellular domains of the neural cell adhesion molecule L1 are involved in homophilic interactions. J. Neurosci. Res. **42**: 9-20.

Horstkorte R., Schachner M., Magyar J.P., Vorherr T., and Schmitz B. (1993). The fourth immunoglobulin-like domain of NCAM contains a carbohydrate recognition domain for oligomannosidic glycans implicated in association with L1 and neurite outgrowth. J. Cell Biol. **121**: 1409-1421.

Hubbe M., Kowitz A., Schirrmacher V., Schachner M., and Altevogt P. (1993). L1 adhesion molecule on mouse leukocytes: Regulation and involvement in endothelial cell binding. Eur. J. Immunol. **23**: 2927-2931.

Ignelzi M.A., Miller D.R., Soriano P., and Maness P.F. (1994). Impaired neurite outgrowth of *src*-minus cerebellar neurones on the cell adhesion molecule L1. Neuron **12**: 873-884.

Imhof B.A., and Dunon D. (1995). Leukocyte migration and adhesion. Advances Immunol. **58**: 345-415.

Kobayashi M., Miura M., Asou H., and Uyemura K. (1991). Molecular cloning of cell adhesion molecule L1 from human nervous tissue: A comparison of the primary sequences of L1 molecules of different origin. Biochim. Biophys. Acta **1090**: 238-240.

Lemmon V., Farr K.L., and Lagenauer C. (1989). L1-mediated axon growth occurs via a homophilic binding mechanism. Neuron **2**: 1597-1603.

Lindner J., Rathjen F.G., and Schachner M. (1983). L1 mono- and polyclonal antibodies modify cell migration in early postnatal mouse cerebellum. Nature **305**: 427-430.

Martini R., and Schachner M. (1986). Immunoelectron microscopic localization of neural cell adhesion molecules (L1, N-CAM, and MAG) and their shared carbohydrate epitope and myelin basic protein in developing sciatic nerve. J. Cell Biol. **103**: 2439-2448.

Miura M., Kobayashi M., Asou H., and Uyemura K. (1991). Molecular cloning of cDNA encoding the rat neural cell adhesion molecule L1. Two L1 isoforms in the cytoplasmic region are produced by differential splicing. FEBS (Fed. Eur. Biochem. Soc.) Lett. **289**: 91-95.

Montgomery A.M.P., Becker J.C., Siu C.-H., Lemmon V.P., Cheresh D.A., Pancook J.D., Zhao X., and Reisfeld R.A. (1996). Human neural cell adhesion molecule L1 and rat homologue NILE are ligands for integrin $\alpha_V\beta_3$. J. Cell Biol. **132**: 475-485.

Needham L.K., and Schnaar R.L. (1993). The HNK-1 reactive sulfoglucuronyl glycolipids are ligands for L-selectin and P-selectin but not E-selectin. Proc. Natl. Acad. Sci. USA **90**: 1359-1363.

Obara M., Kang M.S., and Yamada K.M. (1988). Site-directed mutagenesis of the cell-binding domain of human fibronectin: Separable, synergistic sites mediate adhesive function. Cell **53**: 649-657.

Piali L., Hammel P., Uherek C., Gisler R.H., Dunon D., and Imhof B.A. (1995). CD31/PECAM-1 is a ligand for $\alpha_V\beta_3$ integrin involved in adhesion of leukocytes to endothelium. J. Cell Biol. **130**: 1-10.

Preece G., Murphy G., and Ager A. (1996). Metalloproteinase-mediated regulation of L-selectin levels on leucocytes. J. Biol. Chem. **271**: 11634-11640.

Rathjen F.G., and Schachner M. (1984). Monoclonal antibody L1 recognizes neuronal cell surface glycoproteins mediating cellular adhesion. In Neuroimmunology, Behan P., and Spreafico F., Eds. (New York: Raven Press), pp. 79-87.

Richter-Landsberg C., Lee V.M., Salton S.R.J., Shelanski M.L., and Greene L.A. (1984). Release of the NILE and other glycoproteins from cultured PC12 rat pheochromocytoma cells and sympathetic neurones. J. Neurochem. **43**: 841-848.

Ruoslahti E. (1988). Fibronectin and its receptors. Annu. Rev. Biochem. **57**: 375-413.

Ruppert M., Aigner S., Hubbe M., and Altevogt P. (1995). The L1 adhesion molecule is a cellular ligand for VLA-5. J. Cell Biol. **131**: 1881-1891.

Sammar M., Aigner S., Hubbe M., Schirrmacher V., Schachner M., Vestweber D., and Altevogt P. (1994). Heat-stable antigen (CD24) as ligand for mouse P-selectin. Int. Immunol. **6**: 1027-1036.

Williams A.F. (1987). A year in the life of the immunoglobulin superfamily. Immunol. Today **8**: 298-303.

Zhou H., Fuks A., Alcaraz G., Bolling T.J., and Stanners C.P. (1993). Homophilic adhesion between Ig superfamily carcinoembryonic antigen molecules involves double reciprocal bonds. J. Cell Biol. **122**: 951-960.

